# Transglutaminase 2 at the Interface of Gene Regulation and Antigen Processing in HLA-Restricted Immunity of Celiac Disease

**DOI:** 10.3390/genes17050548

**Published:** 2026-05-03

**Authors:** Faustina Barbara Cannea, Alessandra Padiglia

**Affiliations:** Department of Life and Environmental Sciences (DiSVA), Biomedical Section, University of Cagliari, Cittadella Universitaria di Monserrato, Monserrato, 09042 Cagliari, Italy; faustinab.cannea@unica.it

**Keywords:** celiac disease, transglutaminase 2, TG2, *TGM2*, HLA-DQ2, HLA-DQ8, gluten deamidation, gene regulation, gene environment interaction, autoimmunity

## Abstract

Celiac disease (CD) is an immune-mediated enteropathy triggered by dietary gluten in genetically predisposed individuals. Although HLA-DQ2 and HLA-DQ8 are the primary genetic determinants of susceptibility, they are not sufficient to explain disease onset and progression. A key molecular event in CD pathogenesis is the post-translational modification of gluten peptides by transglutaminase 2 (TG2), which enhances their binding to HLA-DQ molecules and promotes CD4^+^ T cell activation. TG2 also acts as the principal autoantigen, driving the production of anti-TG2 autoantibodies through linked recognition mechanisms. Beyond its enzymatic activity, TG2 is tightly regulated by gene regulatory mechanisms, including cytokine-driven transcription, epigenetic modulation, and stress-responsive signaling pathways. These processes determine TG2 expression and activity in the intestinal mucosa, thereby influencing the efficiency of gluten peptide modification and antigen presentation. Here, we propose that TG2 operates at the interface between gene regulation and antigen processing, linking transcriptional control of *TGM2* to HLA-restricted immune activation. In this framework, disease susceptibility arises from the coordinated interaction between HLA-dependent peptide presentation, TG2-mediated modification of gluten epitopes, and regulation of TG2 expression within the intestinal mucosa. This integrated model provides a mechanistic basis for disease heterogeneity and identifies TG2 as a central regulatory node and potential therapeutic target in CD.

## 1. Introduction

CD is a chronic immune-mediated enteropathy triggered by dietary gluten in genetically predisposed individuals [1,2]. The disease arises from a highly specific interaction between environmental exposure and host immunogenetic background, whereby dietary antigens are recognized within a permissive HLA context. Among the known susceptibility factors, HLA class II molecules, particularly HLA-DQ2 and HLA-DQ8, represent the strongest genetic determinants and are present in the vast majority of affected patients. However, although the HLA genotype is necessary for disease development, it is not sufficient to explain the initiation and amplification of the pathogenic immune response [1,3].

A pivotal molecular event underlying CD pathogenesis is the post-translational modification of gluten-derived peptides mediated by TG2, a multifunctional enzyme widely expressed in epithelial and extracellular compartments. TG2 catalyzes the deamidation of specific glutamine residues within gliadin peptides, converting them into glutamic acid and increasing their negative charge, thereby enhancing binding affinity to HLA-DQ2 and HLA-DQ8 molecules and promoting CD4^+^ T cell activation in the intestinal mucosa [4,5,6,7]. In addition to its enzymatic role, TG2 acts as the principal autoantigen in CD. The formation of TG2-deamidated gliadin complexes contributes to B cell activation and the production of anti-TG2 autoantibodies, hallmark serological markers of the disease [8,9]. Thus, TG2 occupies a dual position in CD pathogenesis, functioning both as an enzymatic amplifier of gluten immunogenicity and as a direct target of the autoimmune response. This dual role indicates that disease susceptibility depends not only on antigen presentation by HLA molecules but also on the regulation of TG2 expression, activity, and conformational state [7,10].

The activity of TG2 is tightly controlled by intracellular calcium levels, guanine nucleotides, and redox conditions, which regulate the equilibrium between its closed (inactive) and open (active) conformations. Inflammatory cytokines and oxidative stress further modulate TG2 expression and catalytic function, linking environmental triggers to molecular regulation. These regulatory layers indicate that CD pathogenesis cannot be fully explained by HLA genetics alone, but instead requires integration of enzymatic, structural, and gene regulatory mechanisms [11,12,13].

In recent years, increasing attention has been directed toward the regulation of *TGM2* expression at multiple levels. Transcriptional control is strongly influenced by inflammatory signaling pathways, particularly those mediated by NF-κB and JAK/STAT, which are activated by cytokines such as IFN-γ and TNF-α. In parallel, epigenetic mechanisms, including DNA methylation, histone modifications, and microRNA-mediated regulation, contribute to fine-tuning *TGM2* expression in response to environmental and inflammatory cues. These regulatory layers dynamically modulate TG2 abundance and activity within the intestinal mucosa.

In contrast, although several polymorphic variants in *TGM2* have been described, their contribution to CD susceptibility appears limited compared with HLA-associated genetic determinants, suggesting that *TGM2* primarily influences disease risk through context-dependent regulatory mechanisms rather than direct genetic predisposition [14]. This distinction suggests that, rather than acting as a primary genetic determinant, *TGM2* may contribute to disease susceptibility through regulated regulatory mechanisms that shape its expression and functional output.

Although the roles of HLA susceptibility and TG2-mediated deamidation have been extensively characterized, these processes are often considered as partially independent components of the pathogenic cascade. However, emerging evidence suggests that TG2 functions as a molecular hub linking environmental gluten exposure to HLA-restricted immune activation [1,7,10]. In this setting, TG2 does not simply modify dietary peptides but integrates enzymatic activity, inflammatory signaling, and gene regulatory mechanisms that collectively shape antigen presentation and immune responsiveness.

This perspective supports a shift from a linear pathogenic model toward a network-based mode in which TG2 operates as a dynamically regulated molecular node embedded within transcriptionally and epigenetically responsive environments. In this model, inflammatory signaling, stress-responsive pathways, and chromatin-level regulation define the conditions under which TG2 expression and activity become permissive for efficient antigen processing and HLA-restricted immune activation.

This review aims to provide an integrated molecular perspective on the role of TG2 in CD by linking enzymatic deamidation, HLA-restricted antigen presentation, autoantigen formation, and gene regulatory mechanisms. The present work moves beyond an enzyme-centered view and proposes TG2 as a gene-regulated integrative node within mucosal immune networks.

By moving beyond static genetic associations and toward dynamically regulated molecular networks, we propose a unified model in which TG2 acts as a central hub connecting environmental exposure to gluten with HLA-dependent adaptive immunity.

Accordingly, this review does not consider TG2-mediated deamidation and HLA-associated susceptibility as isolated pathogenic events, but as functionally integrated components of a broader gene regulatory network. TG2-centered immunity emerges from coordinated interactions between peptide modification, antigen presentation, and gene regulation within the intestinal local tissue environment, ultimately determining when and where pathogenic immune responses are initiated and amplified.

## 2. Structural Organization and Conformational Regulation of TG2

TG2, encoded by the *TGM2* gene located on chromosome 20q12, is a ubiquitously expressed member of the transglutaminase family characterized by marked structural plasticity and functional versatility [15,16]. Unlike other family members with more restricted tissue distribution, TG2 operates both as a Ca^2+^-dependent acyltransferase and as a scaffold protein involved in signal transduction, cell adhesion, and extracellular matrix stabilization. This multifunctionality is tightly linked to its conformational dynamics, which enable TG2 to integrate biochemical signals with cellular and environmental cues. Within the context of CD, these structural properties are not only biochemically regulated but also influenced by gene expression programs controlling TG2 abundance and cellular localization.

### 2.1. Domain Architecture of TG2

Human TG2 is an approximately 77 kDa protein composed of four structurally and functionally distinct domains: an N-terminal β-sandwich domain, a catalytic core domain, and two C-terminal β-barrel domains (β-barrel 1 and β-barrel 2) [16,17].

The N-terminal domain mediates interactions with fibronectin and other extracellular matrix components, facilitating extracellular localization and cell–matrix anchoring. The catalytic core domain contains the active site triad (Cys277, His335, and Asp358), which drives acyl-transfer reactions, including transamidation and deamidation. The two β-barrel domains contribute to intramolecular regulation by controlling access to the catalytic site and stabilizing specific conformational states [16,17].

The spatial organization of these domains directly determines substrate accessibility and catalytic competence. Structural studies have shown that the catalytic cysteine is buried in the inactive conformation and becomes exposed only upon conformational rearrangement, highlighting the central role of structural transitions in regulating TG2 activity [17,18]. Thus, the TG2 structure defines not only enzymatic potential but also the conditions under which antigen-processing activity becomes functionally accessible. These conditions include calcium-dependent conformational activation, which promotes the open and catalytically active state of the enzyme, as well as the local redox environment, which modulates the reactivity of the catalytic cysteine. In addition, substrate availability and accessibility, particularly of gluten-derived peptides in the extracellular space, and the presence of inflammatory signals that enhance TG2 expression and release, collectively determine when and where TG2-mediated antigen processing can occur efficiently.

### 2.2. Open and Closed Conformations: A Molecular Switch

TG2 exists in at least two major conformational states: a closed (compact) GTP-bound form and an open (extended) Ca^2+^-bound form [17,18,19]. These conformations are mutually exclusive and functionally distinct. In the closed conformation, binding of guanine nucleotides (GTP or GDP) stabilizes a compact structure in which the C-terminal β-barrels fold back onto the catalytic core, shielding the active site and rendering the enzyme catalytically inactive [17,19]. In this state, TG2 primarily exerts non-enzymatic functions, including scaffolding and participation in intracellular signaling pathways.

In contrast, elevated intracellular Ca^2+^ levels promote a large conformational rearrangement that displaces the β-barrel domains and exposes the catalytic cysteine, generating the open, enzymatically active conformation [17,18]. This Ca^2+^-dependent transition enables TG2 to catalyze transamidation reactions or, in the absence of suitable amine donors, deamidation reactions relevant to gluten peptide modification in CD.

This conformational switching acts as a molecular integrator of metabolic state, calcium flux, and cellular stress signals. Because intracellular Ca^2+^ levels increase during inflammation and epithelial damage, inflammatory environments favor the catalytically active TG2 conformation, thereby enhancing gluten peptide modification. This mechanism directly links microenvironmental signals to antigen processing, positioning TG2 as a structurally regulated interface between cellular stress responses and HLA-restricted immune activation [7,16].

### 2.3. Catalytic Mechanism: Transamidation Versus Deamidation

The catalytic mechanism of TG2 involves nucleophilic attack by the active-site cysteine on the γ-carboxamide group of a substrate glutamine residue, forming a thioester intermediate [15,16].

At the chemical level, this intermediate represents a key branching point in TG2 catalysis. In transamidation reactions, the thioester intermediate is resolved by reaction with a primary amine donor (R–NH_2_), typically the ε-amino group of a lysine residue, resulting in the formation of an isopeptide bond and release of ammonia (NH_3_) from the glutamine side chain. In contrast, in the absence of suitable amine donors, water acts as the nucleophile in a hydrolytic reaction, leading to hydrolysis of the thioester intermediate and conversion of glutamine into glutamate (deamidation).

These reactions can be summarized as follows for deamidation and transamidation, respectively:

Glutamine (–CONH_2_) → (TG2) → Glutamate (–COO^−^) + NH_3_ (1)

Glutamine (–CONH_2_) + R–NH_2_ → (TG2) → Glutamine–NH–R + NH_3_ (2)

As shown in reactions (1) and (2), the TG2 catalysis diverges depending on the availability of amine donors.

In conditions where suitable amine donors are limited, as commonly occurs in the extracellular environment, the thioester intermediate undergoes hydrolysis, resulting in deamidation and conversion of glutamine residues into glutamic acid [5,20]. This modification introduces a negative charge within gliadin peptides, markedly increasing their affinity for HLA-DQ2 and HLA-DQ8 molecules [5,7,21].

Deamidation is a selective process influenced by local sequence context and peptide conformation. TG2 therefore acts not simply as a catalytic enzyme but as a selective biochemical filter that shapes the repertoire of immunogenic gluten epitopes, directly influencing HLA-restricted antigen presentation and downstream immune activation.

### 2.4. Redox-Dependent Modulation of TG2 Activity

TG2 activity is modulated by cellular redox conditions, which influence the reactivity of the catalytic cysteine (Cys277), a thiol group highly sensitive to oxidative modifications such as S-nitrosylation and disulfide bond formation [22,23].

Redox-dependent regulation of TG2 primarily involves reversible oxidative modifications of this residue. Under oxidizing conditions, Cys277 can undergo disulfide bond formation or S-nitrosylation, leading to inhibition of catalytic activity. Conversely, reducing conditions restore the thiol group, enabling enzymatic function. This redox-sensitive switch provides a dynamic mechanism through which oxidative stress regulates TG2 activity in a context-dependent manner, exerting either inhibitory or modulatory effects depending on the intensity and duration of oxidative conditions [7,22,23].

Extracellular TG2 is particularly sensitive to redox regulation, as disulfide bond formation can stabilize inactive conformations under oxidizing conditions. This sensitivity introduces an additional regulatory layer linking oxidative stress, a hallmark of inflamed intestinal mucosa, to TG2 enzymatic function.

In CD, redox-dependent mechanisms may dynamically regulate TG2 activity across different tissue regions, contributing to spatial and temporal heterogeneity in antigen processing. This variability further supports the concept that TG2 activity is not constitutive but emerges from the integration of biochemical constraints and local microenvironmental signals.

### 2.5. Transcriptional and Post-Translational Regulation of TG2

Beyond conformational control, TG2 expression is regulated at the transcriptional level [16,24]. The *TGM2* gene promoter contains regulatory elements responsive to inflammatory and stress-related signaling pathways, including NF-κB and other transcription factors activated during immune responses [11,25,26]. Pro-inflammatory cytokines such as interferon-γ (IFN-γ) and tumor necrosis factor-α (TNF-α) can upregulate TG2 expression in epithelial and immune cells, increasing enzymatic capacity in inflammatory environments [7,24,27].

Epigenetic mechanisms also contribute to the regulation of *TGM2* expression. DNA methylation, histone modifications, and microRNA-mediated pathways have been shown to modulate TG2 levels in pathological contexts associated with inflammation and tissue remodeling [14,24,25]. In particular, non-coding RNAs may regulate *TGM2* expression at the post-transcriptional level, providing an additional layer of control over enzyme abundance [25].

Post-translational modifications further regulate TG2 function. Phosphorylation, proteolytic cleavage, and alternative splicing can influence TG2 localization, stability, and interactions with intracellular signaling complexes [24]. Although the precise contribution of these mechanisms in CD remains incompletely defined, they likely contribute to the dynamic regulation of TG2 in inflamed intestinal tissues [7].

Taken together, TG2 expression and activity are controlled by a multi-layered regulatory network encompassing transcriptional, epigenetic, and post-translational mechanisms operating within inflammatory environments [7,14,24,28]. In CD, these regulatory layers critically influence local TG2 availability and catalytic activity, thereby modulating the extent of gluten peptide modification and the efficiency of HLA-restricted antigen presentation [7,21,29].

Within this model, TG2 should be interpreted not merely as an enzyme with regulated activity but as a regulated molecular node embedded within gene regulatory networks that integrate inflammatory signaling, cellular stress responses, and microenvironmental cues. This integrative perspective provides a mechanistic link between *TGM2* regulation, enzymatic function, and adaptive immune activation [21,29,30,31].

## 3. TG2-Mediated Deamidation of Gliadin Peptides

Gluten proteins, particularly gliadins and glutenins, are enriched in proline and glutamine residues, which confer resistance to complete proteolytic digestion in the gastrointestinal tract [1,32,33]. As a result, relatively long immunogenic peptides persist within the intestinal lumen and lamina propria [32,33,34]. Under these conditions, TG2-mediated deamidation represents a key biochemical step that selectively reshapes the antigenic properties of gluten peptides, linking protein structure to HLA-restricted immune recognition. This process does not occur in isolation but is embedded within a dynamically regulated intestinal environment that determines the efficiency and extent of antigen processing [7,35,36].

### 3.1. Substrate Specificity and Sequence Context

TG2 activity is highly selective and strongly influenced by local amino acid sequences and structural accessibility. Glutamine residues within specific sequence contexts, often enriched in proline, are preferentially targeted [37]. The high proline content of gliadin peptides not only limits proteolytic degradation but also promotes conformations favorable for TG2 recognition, thereby coupling resistance to digestion with enzymatic susceptibility [38]. The repetitive nature of gliadin sequences generates multiple TG2-sensitive motifs within individual peptides, increasing the likelihood of deamidation events and enabling the generation of multiple modified epitopes from a single parent sequence [32,39,40].

Deamidation typically occurs at defined positions within immunodominant epitopes, converting neutral glutamine residues into negatively charged glutamic acid residues. This seemingly minimal chemical modification has major functional consequences, enhancing peptide compatibility with HLA molecules and stabilizing T cell receptor engagement within a genetically restricted context [1,7,41]. Accordingly, substrate specificity represents a key determinant of how biochemical processing is translated into immunological recognition.

### 3.2. Chemical Consequences of Glutamine-to-Glutamate Conversion

TG2-mediated deamidation introduces negatively charged residues into gliadin peptides, altering electrostatic interactions and local peptide conformation [1,7]. In CD, this modification selectively increases the affinity of gliadin-derived epitopes for HLA-DQ2 and HLA-DQ8 molecules, promoting the formation of stable peptide–MHC complexes [1,14]. TG2 acts as a biochemical amplifier, converting weakly immunogenic peptides into high-affinity ligands capable of efficient antigen presentation. Increased peptide–MHC stability prolongs surface display and supports sustained T cell receptor engagement, thereby enhancing downstream adaptive immune responses [1,38,42]. This amplification process reflects not only intrinsic enzymatic activity but also the regulatory conditions that govern TG2 availability and activation within the intestinal mucosa.

### 3.3. Generation of Neo-Epitopes and Immunodominance

Deamidation not only enhances binding affinity but also contributes to the generation of neo-epitopes [1,7]. Peptides with limited immunogenicity in their native form can acquire strong antigenic potential following TG2-mediated modification, indicating that TG2 actively reshapes the antigenic landscape rather than merely amplifying pre-existing signals.

Several immunodominant epitopes identified in CD are preferentially recognized in their deamidated form. T cells isolated from the intestinal mucosa of affected individuals typically exhibit stronger responses to deamidated peptides compared with their native counterparts [36,43], supporting the view that TG2 activity is required for efficient adaptive immune activation [10].

The multivalent nature of gliadin peptides further enables the formation of TG2–gliadin complexes, providing a mechanistic basis for linked recognition. In this process, TG2-specific B cells internalize the complex and present gliadin-derived peptides to gluten-reactive T cells, promoting cooperation between B and T lymphocytes [8,9]. This interaction represents a critical step in the generation of anti-TG2 autoantibodies [30]. From this perspective, TG2-mediated modification not only enhances antigen presentation but also contributes to the coordination of adaptive immune responses across cellular compartments.

### 3.4. Spatial and Temporal Regulation of Deamidation in the Intestinal Mucosa

TG2-mediated deamidation occurs primarily in the extracellular environment of the intestinal lamina propria, where elevated calcium concentrations and inflammatory signals favor the catalytically active conformation of the enzyme [44,45]. However, TG2-mediated deamidation may also occur in intestinal epithelial cells, particularly under inflammatory conditions, as suggested by recent studies, demonstrating that epithelial TG2 can contribute to local antigen processing [46]. Tissue damage, epithelial stress, and increased intestinal permeability facilitate the translocation of gliadin peptides across the epithelial barrier, allowing access to TG2 in the lamina propria. In addition, immunoglobulin A from patients with CD may contribute to epithelial transport and local handling of gliadin peptides, increasing their availability for TG2-mediated modification [47]. Inflammatory cytokines further enhance TG2 expression in epithelial and immune cells, increasing local enzymatic capacity. Consequently, deamidation represents a regulated process amplified under inflammatory conditions rather than a constitutive event [48,49].

This dynamic regulation highlights the interplay between genetic predisposition and the local biochemical environment. Individuals carrying permissive HLA haplotypes may remain asymptomatic in the absence of sufficient TG2 activation or inflammatory stimuli, underscoring the role of environmental and regulatory factors in disease onset [50].

TG2-mediated deamidation shapes the repertoire of gluten-derived peptides presented by HLA-DQ molecules, thereby linking environmental antigen exposure to adaptive immune activation. In this model, TG2 should be viewed not simply as an enzyme that modifies dietary proteins, but as a regulated enzymatic filter whose activity is determined by microenvironmental cues and gene regulatory mechanisms. By integrating substrate structure, inflammatory signaling, and transcriptional control, TG2 defines the efficiency and threshold of CD4^+^ T cell activation [51]. Consequently, the extent of TG2-mediated deamidation depends not only on substrate properties but also on transcriptional regulation of *TGM2* expression in inflamed tissues.

## 4. HLA-Restricted Presentation of Deamidated Gliadin Peptides

The genetic predisposition to CD is primarily determined by HLA-DQ2 and HLA-DQ8 molecules, which provide the structural basis for antigen presentation to CD4^+^ T cells. However, HLA molecules do not initiate immune activation on their own but act as selective platforms whose immunological outcome depends on the biochemical properties of presented peptides and the regulatory context in which antigen presentation occurs. In this integrative view, TG2-mediated modification of gluten peptides represents the critical interface through which environmental antigen exposure is translated into HLA-restricted immune recognition. The pathogenic cascade therefore arises from the coordinated interaction between enzymatic peptide modification, HLA-dependent antigen presentation, and microenvironment-dependent regulatory processes, including cytokine signaling, epithelial barrier integrity, extracellular calcium availability, and redox conditions within the intestinal mucosa [52].

### 4.1. Enhanced Binding of Deamidated Peptides to HLA-DQ2 and HLA-DQ8

HLA-DQ2 and HLA-DQ8 exhibit binding preferences that favor negatively charged residues at specific anchor positions within presented peptides. Native gliadin peptides generally display suboptimal affinity for these HLA molecules, whereas TG2-mediated deamidation introduces glutamic acid residues that increase electrostatic compatibility with the peptide-binding groove. Structural studies have shown that negatively charged residues are preferentially accommodated at defined anchor positions, such as P4, P6, and P7, depending on the HLA haplotype, thereby stabilizing peptide–HLA interactions and promoting immunodominance. Rather than acting independently, HLA binding specificity and TG2-mediated deamidation operate as tightly coupled processes that determine peptide selection, stability, and immunogenic potential.

Antigen recognition can be conceptualized as a two-step process in which genetic predisposition establishes a permissive HLA context, while TG2-mediated deamidation converts weak ligands into high-affinity immunogenic peptides [21,52,53]. Recent structural and in silico analyses further support the concept that HLA-DQ-associated susceptibility is driven by fine molecular interactions between peptide residues and specific binding pockets within the HLA groove. In particular, negatively charged residues introduced by TG2-mediated deamidation are preferentially accommodated at defined anchor positions (e.g., P4, P6, or P7), where they establish stabilizing electrostatic interactions with positively charged residues in the HLA binding groove. These interactions increase peptide–HLA stability and enhance T cell receptor recognition, thereby promoting immunodominance [54].

### 4.2. Functional Amplification of T Cell Responses

Stabilization of peptide–HLA complexes enables efficient activation of gluten-specific CD4^+^ T helper cells in the intestinal lamina propria. These T cells predominantly exhibit a Th1 phenotype, characterized by production and contribution to mucosal inflammation.

Activated T cells also provide help to TG2-specific B cells through recognition of TG2–gliadin complexes, promoting class switching and the production of anti-TG2 autoantibodies. Within this regulatory landscape, HLA restriction not only enables antigen presentation but also shapes the magnitude and quality of the adaptive immune response. The density and persistence of peptide–HLA complexes directly reflect the efficiency of TG2-mediated peptide modification, thereby linking enzymatic processing to the strength of T cell activation. Sustained gluten exposure maintains this pathogenic loop through continuous coupling between TG2 activity and HLA-dependent antigen presentation [55,56,57]. The magnitude and persistence of the immune response are strongly influenced by the amount and duration of gluten ingestion, with continuous dietary exposure sustaining antigen presentation and T cell activation. The intensity of this response is further modulated by the intestinal microenvironment, which governs both TG2 expression and antigen-presenting cell activation. Key elements include pro-inflammatory cytokines such as IFN-γ, TNF-α, IL-15, and IL-1β, which enhance *TGM2* transcription and promote immune activation. In addition, increased intestinal permeability facilitates gliadin translocation, while elevated extracellular calcium levels support TG2 activation. Redox imbalance and epithelial stress further contribute to shaping the local environment, collectively influencing antigen processing and the activation of antigen-presenting cells.

### 4.3. HLA Genotype as a Context-Dependent Determinant of Immunogenicity

Although HLA-DQ2 and HLA-DQ8 molecules are required for disease development, their presence alone does not inevitably result in autoimmunity. These molecules are encoded by specific *HLA-DQA1* and *HLA-DQB1* alleles, most commonly forming the HLA-DQ2.5 heterodimer (HLA-DQA1*05:01/HLA-DQB1*02:01) or the HLA-DQ8 heterodimer (HLA-DQA1*03:01/HLA-DQB1*03:02), which exhibit high binding affinity for deamidated gluten peptides. Disease risk is further influenced by gene dosage effects at the HLA-DQ locus [58,59]. Individuals carrying two copies of the HLA-DQB1*02 allele exhibit a significantly higher risk of CD, likely due to increased density of HLA-DQ molecules capable of presenting deamidated peptides. However, a substantial proportion of individuals carrying these haplotypes remain asymptomatic, highlighting the importance of additional modulatory factors, including TG2 activation, inflammatory microenvironment, epithelial barrier integrity, and gene regulatory mechanisms [60,61].

The HLA genotype therefore defines a permissive background rather than a deterministic outcome. Disease onset occurs only when genetic susceptibility is coupled with efficient TG2-mediated peptide modification and a permissive inflammatory and transcriptionally active environment. In this integrated model, HLA molecules provide the structural constraints for antigen presentation, whereas TG2 activity determines the quality and availability of immunogenic ligands, and gene regulatory networks define the conditions under which this interaction becomes pathogenic [10,52,62,63].

This perspective reconciles the high prevalence of HLA-DQ2/DQ8 haplotypes in the general population with the lower incidence of overt CD, indicating that immune activation is a conditional process emerging from the interplay between genetic predisposition, enzymatic peptide modification, and regulated regulatory networks operating within the intestinal microenvironment [62,63,64].

## 5. TG2 as an Autoantigen and Epitope Spreading

While TG2 plays a pivotal enzymatic role in modifying gluten peptides, it also represents the principal autoantigen in CD. The development of anti-TG2 autoantibodies reflects a breakdown of tolerance toward this ubiquitously expressed self-protein. This dual role positions TG2 at the center of the pathogenic cascade, where enzymatic activity, antigen presentation, and adaptive immune responses converge within a coordinated and dynamically regulated system.

### 5.1. Formation of TG2–Gliadin Complexes and Linked Recognition

A key mechanistic feature of CD pathogenesis is the formation of covalent complexes between TG2 and gliadin peptides [7]. During transamidation reactions, TG2 forms stable enzyme–substrate intermediates with gluten-derived peptides, generating molecular structures that physically couple a self-protein (TG2) with a foreign antigen (gliadin). This coupling enables linked recognition, a process in which TG2-specific B cells bind TG2–gliadin complexes via their B cell receptor, internalize them, and process the gliadin component, including TG2-modified peptides [8,64]. The resulting peptides, typically in their deamidated form, are presented on HLA-DQ2 or HLA-DQ8 molecules to gluten-specific CD4^+^ T cells, which in turn provide cognate help to TG2-reactive B cells.

This mechanism establishes a functional bridge between TG2 enzymatic activity and adaptive immune activation. Rather than requiring TG2-specific T cell priming, gluten-specific T cells indirectly orchestrate the autoimmune response by supporting TG2-specific B cells, thereby coupling antigen processing to autoantibody production. In this setting, TG2-mediated modification operates as a regulatory checkpoint that enables coordinated interactions between antigen presentation and B cell activation.

### 5.2. Autoantibody Production and Diagnostic Relevance

Anti-TG2 antibodies, particularly of the IgA isotype, are highly sensitive and specific for CD and form the basis of modern serological diagnosis. Their presence reflects ongoing immune activation within the intestinal mucosa and correlates with gluten exposure. Autoantibody production is antigen-driven and dependent on continuous gluten intake. Removal of dietary gluten leads to a progressive decline in antibody titers, highlighting the reversible nature of the autoimmune response and its dependence on continuous antigen exposure.

Mechanistically, anti-TG2 antibodies are generated through germinal center reactions within gut-associated lymphoid tissue, where sustained T–B cell interactions promote affinity maturation and class switching. The relatively restricted antibody repertoire observed in patients supports antigen-driven clonal selection rather than non-specific polyclonal activation [42,65]. The efficiency of these processes depends on the broader regulatory environment that governs antigen availability, TG2 expression, and T cell help within the intestinal microenvironment.

### 5.3. Epitope Spreading and Amplification of Autoimmunity

The formation of TG2–gliadin complexes also facilitates epitope spreading. Initially, immune responses target specific deamidated gluten epitopes; however, antigen recognition progressively expands to additional gliadin epitopes and multiple regions within the TG2 molecule.

This diversification broadens both B cell and T cell repertoires, reinforcing the autoimmune response and contributing to sustained inflammation. The coexistence of antibodies against native gliadin, deamidated gliadin peptides, TG2, and endomysial antigens reflects this layered immune activation [7,64].

Collectively, these mechanisms indicate that TG2 not only initiates but also amplifies autoimmunity by promoting diversification of antigenic targets within a context of persistent immune stimulation. This expansion of immune specificity can be interpreted as a dynamic process shaped by continuous antigen processing and regulatory feedback within the mucosal immune environment.

### 5.4. Context-Dependent Breakdown of Tolerance

Despite its ubiquitous expression, TG2 becomes an autoimmune target only under specific conditions. Loss of tolerance requires the convergence of multiple factors, including permissive HLA genotype, TG2-mediated peptide modification, intestinal inflammation, and sustained antigen exposure.

Under these conditions, TG2 enzymatic activity generates immunogenic complexes within an inflammatory microenvironment, enabling efficient HLA-restricted antigen presentation and activation of adaptive immune responses. Tolerance breakdown is not a static event but a regulated process emerging from the interaction between antigen availability, immune activation, and gene regulatory mechanisms controlling TG2 expression and function.

Under these conditions, TG2 functions as a regulated molecular node in which enzymatic processing, antigen presentation, and immune activation are functionally integrated. This process transforms a physiological protein into a conditional autoantigen capable of sustaining immune responses only when biochemical, genetic, and regulatory signals converge [8]. These observations support a model in which TG2 links enzymatic peptide modification to adaptive immune responses within a dynamically regulated inflammatory and transcriptionally responsive context, further reinforcing its role as a central integrator of TG2-centered immunity.

## 6. Genetic Regulation of the TGM2 Gene

Beyond its biochemical regulation, TG2 must be interpreted within the regulatory logic of the *TGM2* gene itself. Inflammatory cytokines, epithelial stress pathways, chromatin remodeling, and non-coding RNA networks collectively shape the transcriptional landscape that enables TG2-centered immunity.

While TG2 activity is classically controlled at conformational and biochemical levels, increasing evidence indicates that transcriptional, epigenetic, and genetic mechanisms critically determine its expression. Rather than acting as a constitutively expressed enzyme, TG2 should be viewed as a dynamically regulated molecular node tightly coupled to inflammatory signaling and cellular stress responses. Variations in TG2 abundance across tissues and pathological conditions directly influence local enzyme availability and the extent of gluten peptide modification within the intestinal mucosa.

### 6.1. Genomic Organization and Promoter Structure

The *TGM2* gene, located on chromosome 20q12, comprises multiple exons that can generate alternative splice variants with distinct functional properties [66]. Its promoter region contains binding sites for transcription factors involved in inflammatory and stress-responsive pathways, including NF-κB, AP-1, and STAT family members [67,68,69].

Under basal conditions, *TGM2* transcription is tightly regulated and cell-type-specific [70]. However, exposure to pro-inflammatory cytokines such as INF-γ, TNF-α, and interleukin-1β (IL-1β) can markedly enhance transcription in epithelial and immune cells. These signals are particularly relevant in the intestinal mucosa of patients with CD, where cytokine production is sustained during gluten exposure [71,72].

This responsiveness indicates that TG2 expression is dynamically coupled to mucosal immune activation, increasing enzymatic capacity in the microenvironment where gluten peptides are encountered [7]. In this sense, promoter architecture enables rapid transcriptional responsiveness to inflammatory stimuli, linking gene structure to the regulation of TG2 expression.

### 6.2. Cytokine-Driven Regulation of TGM2 Expression

Cytokine-mediated signaling, driven by pro-inflammatory cytokines such as IFN-γ, TNF-α, IL-15, and IL-1β, represents a central regulatory axis controlling *TGM2* transcription within the intestinal mucosa. Rather than being governed by individual mediators, *TGM2* expression emerges from the integration of a coordinated pro-inflammatory cytokine network that links innate immune activation, epithelial stress responses, and adaptive immunity [52].

Among the key regulators, IFN-γ, TNF-α, and IL-1β act as primary drivers of inflammatory transcriptional programs. These cytokines activate canonical signaling pathways, including NF-κB and JAK/STAT cascades, which converge on the *TGM2* promoter and enhance its transcriptional activity in both epithelial and immune cells [73]. In particular, IFN-γ- and TNF-α-dependent signaling has been shown to synergistically upregulate *TGM2* expression in intestinal tissues, directly linking inflammatory cytokine exposure to increased TG2 availability [71].

TG2 is not merely a downstream effector of inflammation but also participates in reinforcing inflammatory signaling. Experimental evidence indicates that TG2 can modulate NF-κB activity and contribute to cytokine-driven signaling pathways, thereby establishing a positive feedback loop that sustains inflammatory responses [70,73]. This bidirectional interaction positions TG2 as both a target and an active regulator within the inflammatory network.

Beyond these classical mediators, interleukin-15 (IL-15) has emerged as a critical upstream regulator of mucosal immune activation. IL-15 is primarily produced by intestinal epithelial cells and antigen-presenting cells (APCs) in response to gliadin peptide exposure and cellular stress, and plays a central role in shaping the epithelial–immune interface [52]. IL-15-driven signaling promotes the activation, survival, and cytotoxic differentiation of intraepithelial lymphocytes (IELs), thereby lowering the threshold for immune activation and enabling antigen-independent cytotoxic responses [21].

In the intestinal mucosa of patients with CD, IL-15-mediated activation of IELs induces the expression of activating receptors such as natural killer group 2, member D (NKG2D) and enhances epithelial cell killing, leading to barrier disruption and increased antigen translocation [21]. This process amplifies local inflammation and synergizes with IFN-γ-mediated responses, contributing to sustained activation of NF-κB-dependent transcriptional programs and indirectly promoting *TGM2* expression [52,73].

In parallel, gliadin-induced cellular stress responses further amplify cytokine-driven regulation of *TGM2*. Activation of the unfolded protein response (UPR) in intestinal epithelial cells leads to increased expression of TG2 and pro-inflammatory cytokines, establishing a direct mechanistic link between environmental antigen exposure, epithelial stress, and inflammatory signaling cascades [74].

Collectively, these pathways define a feed-forward regulatory circuit in which cytokine signaling, epithelial stress responses, and TG2 activity are tightly interconnected [21,52]. Pro-inflammatory cytokines upregulate *TGM2* transcription, increasing TG2 availability for deamidation of gliadin peptides, which in turn enhances antigen presentation and adaptive immune activation. This integrated network transforms *TGM2* from a passive enzymatic component into an active amplifier of mucosal inflammation, driving the persistence and escalation of the pathogenic immune response in CD.

### 6.3. Epigenetic and Post-Transcriptional Regulation

Epigenetic mechanisms provide an additional layer of control over gene expression independently of DNA sequence variation. In CD, these processes shape transcriptional programs within the intestinal mucosa in response to environmental stimuli, particularly chronic gluten exposure and inflammation. Key mechanisms include DNA methylation, histone modifications, and regulatory non-coding RNAs such as microRNAs [75,76,77].

Alterations in DNA methylation patterns have been reported in intestinal epithelial and immune cells of patients with active disease, affecting genes involved in immune regulation, epithelial barrier function, and inflammatory responses [78]. These changes influence promoter activity and transcription factor accessibility, contributing to sustained activation of immune-related pathways.

Histone modifications represent an additional level of chromatin control. Post-translational modifications such as histone acetylation and methylation regulate chromatin accessibility and transcriptional activity. In inflammatory conditions, increased histone acetylation is generally associated with transcriptional activation of immune-related genes, whereas histone methylation can exert either activating or repressive effects, depending on the specific residues involved [79,80,81].

Regulatory non-coding RNAs, including microRNAs and long non-coding RNAs (lncRNAs), further contribute to the fine-tuning of gene expression. MicroRNAs such as miR-21, miR-155, and miR-31 regulate mRNA stability and translation of genes involved in immune responses and epithelial barrier function [82]. In addition, emerging evidence suggests that lncRNAs can modulate chromatin structure and transcriptional programs by interacting with transcription factors and epigenetic modifiers, thereby contributing to context-dependent gene expression in the intestinal mucosa.

Although direct epigenetic regulation of *TGM2* has not been fully characterized, these mechanisms likely influence its expression indirectly by modulating upstream transcriptional pathways. Overall, epigenetic and post-transcriptional processes represent a dynamic interface through which environmental exposure is translated into sustained alterations of mucosal gene expression, embedding TG2 activity within broader regulatory networks.

### 6.4. Genetic Variability and Polymorphisms in TGM2

In contrast to the strong association observed at the HLA locus, polymorphisms within *TGM2* have not been identified as major determinants of CD susceptibility. Genome-wide association studies have consistently highlighted immune-related loci outside the *TGM2* region, indicating that this gene does not represent a primary genetic driver of disease risk.

Nevertheless, several SNPs and regulatory variants have been described within *TGM2*, including variants located in promoter regions, introns, and untranslated regions that may influence transcriptional activity, splicing efficiency, or mRNA stability [83].

Variants such as rs2076380 and rs4811528 have been reported within regulatory regions and may affect transcription factor binding or responsiveness to inflammatory signaling. Although these variants do not confer high genetic risk, they may modulate TG2 expression levels and enzymatic availability in the intestinal mucosa [84].

Thus, *TGM2* variability likely contributes to disease susceptibility through subtle regulatory effects rather than structural alterations of the protein. Even modest changes in transcriptional responsiveness may influence the local threshold required for efficient deamidation of gliadin peptides and antigen presentation within a permissive HLA background.

### 6.5. Non-HLA Genetic Variants and Regulatory Networks in CD

Beyond the HLA locus, the genetic architecture of CD includes numerous non-HLA susceptibility loci involved in immune regulation, T cell activation, and cytokine signaling pathways. These findings indicate that disease susceptibility is largely driven by perturbations in immune regulatory networks rather than by single gene effects [85].

Key loci include genes such as *IL2*, *IL21*, and *SH2B3*, which regulate T cell proliferation and cytokine signaling, as well as genes involved in lymphocyte activation and immune synapse organization, including *TAGAP* and *CTLA4*. A substantial proportion of these variants are located in non-coding regions and exert their effects through modulation of gene expression, enhancer activity, and chromatin accessibility rather than through structural alterations of encoded proteins [86].

Functional analyses, including gene expression profiling, transcription factor binding studies, and experimental validation assays, indicate that many risk variants influence transcription factor binding and regulatory element activity, shaping cell-type-specific transcriptional programs within the intestinal immune environment.

Genetic susceptibility therefore emerges from coordinated alterations in gene expression networks controlling immune activation and tolerance [85].

TG2-mediated peptide modification operates as a biochemical amplifier embedded within these systems. The interaction between regulatory genetic variants, HLA-dependent antigen presentation, and TG2-mediated processing determines the efficiency of gluten-specific CD4^+^ T cell activation and the magnitude of adaptive immune responses [6,38,54].

Collectively, these findings support a model in which TG2 expression and activity are not fixed properties but arise from the coordination of genetic, transcriptional, and environmental signals. TG2 acts as a central molecular node linking gene expression control to antigen processing and immune activation, thereby providing a mechanistic basis for disease heterogeneity.

To summarize the multi-layered regulatory mechanisms controlling TG2 activity, the main pathways are integrated in Table 1.

Major non-HLA susceptibility loci and their functional roles in immune regulation are summarized in Table 2.

### 6.6. Integration of Gene Regulation, Disease Susceptibility, and Gene–Environment Interaction

Building on this regulatory perspective, *TGM2* expression is dynamically shaped by inflammatory and stress-responsive pathways that are activated upon gluten exposure [86]. Immune activation triggers cytokine signaling cascades that upregulate *TGM2* transcription, increase TG2 availability, and amplify deamidation of gliadin peptides [7,41,64,74,87].

Disease susceptibility in CD represents a paradigmatic example of gene–environment interaction in human autoimmunity. Disease development requires the convergence of genetic predisposition, primarily conferred by HLA-DQ2 or HLA-DQ8 haplotypes, and environmental exposure to dietary gluten. However, genetic susceptibility alone is insufficient, as many individuals remain asymptomatic throughout life. Environmental exposure must therefore intersect with additional factors that modulate immune responsiveness, including mucosal inflammation, epithelial barrier integrity, and regulatory signaling pathways [88,89].

Within this context, TG2-mediated peptide modification acts as a molecular interface through which environmental antigen exposure is translated into HLA-restricted immune activation. By converting gluten-derived peptides into high-affinity ligands for HLA-DQ molecules, TG2 amplifies the immunogenic potential of dietary proteins within a genetically defined immune landscape [90]. Overall, disease susceptibility emerges from the coordinated interaction between HLA-mediated peptide presentation, TG2-driven biochemical modification, and transcriptional regulation controlling enzyme availability. Non-HLA susceptibility loci further modulate immune signaling pathways and influence the threshold required for efficient antigen presentation and T cell activation [88]. This integrated model provides a mechanistic basis for inter-individual variability among carriers of permissive HLA haplotypes.

### 6.7. Transcriptomic Signatures of Intestinal Mucosa in CD

Transcriptomic analyses have revealed coordinated activation of inflammatory, epithelial stress, and antigen presentation pathways in the intestinal mucosa of patients with CD [91]. In particular, these studies highlight the upregulation of interferon-responsive genes, antigen presentation pathways, and pro-inflammatory signaling networks. RNA sequencing studies consistently show upregulation of interferon signaling, cytokine-mediated responses, and antigen-processing pathways, reflecting the activation of gluten-specific CD4^+^ T cells and the establishment of a pro-inflammatory microenvironment [41,91]. Beyond descriptive profiling, these transcriptional signatures provide functional insight into the transcriptional, epigenetic, and inflammatory regulatory context in which TG2 operates. In particular, the coordinated induction of interferon-responsive genes and antigen presentation machinery defines a transcriptional landscape that promotes efficient peptide processing and immune activation.

These data indicate that TG2 expression and activity are not uniformly distributed but are embedded within specific transcriptionally active niches of the intestinal mucosa. TG2-mediated peptide modification is selectively amplified in regions characterized by high inflammatory and antigen-processing activity [38]. Thus, transcriptomic data support a model in which TG2-dependent enzymatic activity is functionally integrated into broader gene expression programs coordinating epithelial stress responses, immune activation, and antigen presentation.

### 6.8. Single-Cell Transcriptomic Insights into the Celiac Intestinal Microenvironment

Single-cell RNA sequencing has further refined the understanding of cellular networks in CD by resolving cell-type-specific transcriptional programs within the intestinal mucosa. These analyses reveal extensive remodeling of immune and epithelial cell populations, including expansion of activated CD4^+^ T cells, cytotoxic IELs, and antigen-presenting myeloid cells [92].

At the epithelial level, single-cell approaches have identified distinct transcriptional states characterized by interferon-responsive gene expression, stress signaling, and enhanced antigen-processing capacity. These findings highlight the active role of epithelial cells as dynamic participants in immune regulation rather than passive targets of inflammation.

Single-cell data suggest that TG2 activity is preferentially associated with specific cellular niches in which inflammatory signaling, epithelial stress, and antigen presentation converge [93]. In these microenvironments, TG2-mediated peptide modification acts as a biochemical trigger interfacing with cell-type-specific transcriptional programs and amplifying immune activation.

This spatially and cellularly resolved view indicates that TG2 function is tightly coupled to the organization of the intestinal immune microenvironment. Rather than acting uniformly across tissues, TG2 activity is shaped by local cellular states and signaling pathways that define the threshold for antigen presentation and adaptive immune responses [21,22].

**Figure 1:**
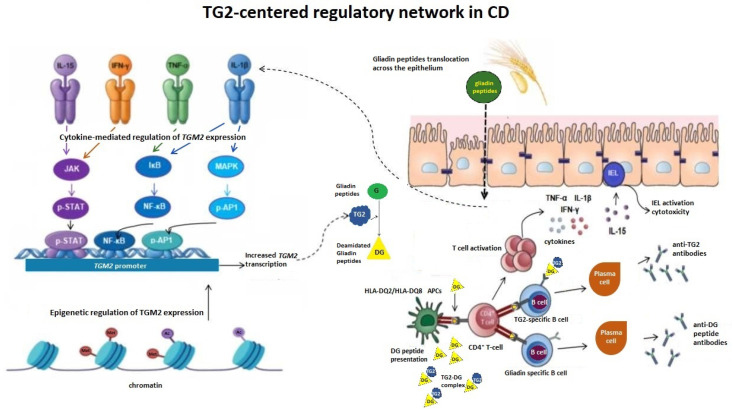
TG2 links gene regulation to antigen processing and HLA-restricted immunity in CD. Pro-inflammatory cytokines, including IL-15, IFN-γ, TNF-α, and IL-1β, activate intracellular signaling pathways such as JAK/STAT, NF-κB, and MAPK, leading to transcriptional upregulation of the *TGM2* gene. Epigenetic mechanisms, including chromatin remodeling, further modulate *TGM2* expression and contribute to context-dependent regulation. In the intestinal mucosa, gluten-derived gliadin peptides translocate across the epithelial barrier and are selectively deamidated (DG) by TG2, generating negatively charged epitopes with increased affinity for HLA-DQ2/DQ8 molecules on APCs. This promotes the activation of gluten-specific CD4^+^ T cells and the release of pro-inflammatory cytokines. TG2-deamidated gliadin (TG2-DG) complexes are internalized by TG2-specific B cells, enabling linked recognition and T cell-dependent B cell activation, leading to the production of anti-TG2 and anti-DG peptide antibodies by plasma cells. In parallel, IL-15-driven activation of IELs enhances epithelial cytotoxicity and barrier disruption, facilitating further gliadin translocation. These processes establish a feed-forward loop in which cytokine signaling, *TGM2* gene regulation, TG2 enzymatic activity, and adaptive immune responses reinforce each other, sustaining chronic intestinal inflammation.

The TG2-centered regulatory network linking cytokine-driven gene expression, gliadin modification, and immune activation is illustrated in Figure 1.

## 7. Redox and Inflammatory Modulation of TG2 in the Intestinal Microenvironment

The intestinal mucosa of patients with CD is characterized by a dynamic inflammatory microenvironment in which cytokine production, epithelial stress, oxidative imbalance, and barrier dysfunction coexist. Within this context, TG2 activity is tightly regulated by local biochemical and inflammatory conditions, including pro-inflammatory cytokines, redox status, extracellular calcium levels, and epithelial barrier integrity, which together determine the extent of gluten peptide modification and downstream immune activation.

### 7.1. Oxidative Stress and Redox-Sensitive Regulation of TG2

Oxidative stress is a well-established feature of active CD, with increased levels of reactive oxygen species (ROS) detected in the intestinal mucosa [94]. TG2 contains a catalytic cysteine residue (Cys277) that is highly sensitive to redox modifications. Oxidation of this thiol group can transiently inhibit catalytic activity through reversible disulfide bond formation or S-nitrosylation, whereas reducing conditions restore enzymatic competence [95,96].

This redox sensitivity introduces a regulatory mechanism linking oxidative balance to antigen processing. Under conditions of moderate inflammation, transient oxidative modifications may fine-tune TG2 activity through redox-dependent mechanisms [97]. In contrast, sustained oxidative stress can disrupt the equilibrium between inactive and active TG2 conformations, contributing to spatial and temporal heterogeneity in deamidation of gliadin peptides within the intestinal mucosa [22].

Moreover, local antioxidant capacity, particularly glutathione availability, may influence TG2 enzymatic output. Variations in redox buffering systems may therefore modulate the threshold required for efficient gliadin modification and subsequent adaptive immune activation. In this sense, redox regulation acts as a dynamic filter that determines when TG2 activity becomes permissive for effective antigen processing.

### 7.2. Cytokine-Mediated Amplification of TG2 Activity

Inflammatory cascade triggered by gluten ingestion leads to the production of cytokines such as IFN-γ, TNF-α, and IL-15. While these mediators enhance *TGM2* expression at the transcriptional level, as discussed in Section 6.2, they also modulate TG2 activity indirectly by altering the local tissue environment.

In particular, cytokine-driven epithelial damage and increased intestinal permeability promote extracellular calcium accumulation, which favors the open, catalytically active conformation of TG2 [7]. In this way, inflammatory signaling is directly coupled to enhanced enzymatic activity [71].

This interaction establishes a feed-forward loop in which inflammation increases TG2 activity and TG2 activity enhances the generation of immunogenic gluten-derived peptides. The resulting amplification of antigen presentation contributes to the persistence of mucosal immune activation in the presence of dietary gluten. From this perspective, cytokine signaling acts not only as an upstream trigger but also as a regulatory driver that integrates transcriptional induction and enzymatic activation of TG2.

### 7.3. Barrier Dysfunction and Spatial Regulation of TG2 Activity

Increased intestinal permeability is a well-recognized feature of CD and facilitates the translocation of partially digested gluten peptides into the lamina propria. Barrier dysfunction therefore exposes these peptides to extracellular TG2 within a microenvironment enriched in calcium and inflammatory mediators [44].

The spatial distribution of TG2 is a key determinant of peptide modification efficiency. Under physiological conditions, TG2 contributes to extracellular matrix stabilization and tissue repair [98]. During inflammation, however, its functional role shifts toward enhanced deamidation of infiltrating gliadin peptides, thereby increasing their immunogenicity [7]. Epithelial stress and mechanical damage may further promote TG2 release from cells, increasing the extracellular pool of active enzyme. This redistribution enables localized amplification of immunogenic peptide generation at sites of mucosal injury, linking barrier disruption to enhanced antigen processing within defined microanatomical compartments.

Significantly, this spatial organization indicates that TG2 activity is not uniformly distributed but is confined to specific microenvironments in which inflammatory signaling, substrate availability, and regulatory cues converge.

### 7.4. Integration of Redox, Inflammation, and Genetic Predisposition

The interplay between redox balance, inflammatory signaling, and HLA genotype provides a conceptual basis for understanding disease heterogeneity. Individuals carrying permissive HLA-DQ haplotypes may remain asymptomatic when TG2 activation is constrained by controlled inflammatory responses or effective redox buffering. Conversely, sustained inflammatory conditions can enhance TG2 expression and activity, promoting sufficient deamidation of gliadin peptides to sustain pathogenic T cell activation [95,96,99]. TG2 operates as a regulated integrator of environmental exposure and host regulatory networks, where its activity reflects the convergence of structural permissiveness, transcriptional regulation, and microenvironmental cues. This integrative view reinforces the concept that TG2-centered immunity emerges from the coordinated interaction between gene regulatory programs and local biochemical conditions.

This perspective highlights that CD is not solely determined by genetic predisposition but arises from dynamic interactions between biochemical regulation, gene expression, and immune activation within the intestinal microenvironment.

### 7.5. Microbiota and Environmental Modulation of Intestinal Immunity

Increasing evidence suggests that the intestinal microbiota contributes to shaping the mucosal immune environment in CD. Alterations in microbial composition (dysbiosis) have been reported in genetically predisposed individuals and may influence epithelial barrier integrity, antigen processing, and local immune responses. Microbial metabolites, including short-chain fatty acids (SCFAs), as well as microbial-derived inflammatory signals, can modulate epithelial permeability, cytokine production, and redox balance within the intestinal mucosa. These changes may indirectly regulate TG2 activity by affecting substrate accessibility, calcium-dependent activation, and the inflammatory milieu in which gliadin peptides are processed [100].

Although the precise causal relationships remain under investigation, microbiota-dependent modulation of mucosal immunity represents an additional environmental layer capable of influencing disease susceptibility and progression.

## 8. Therapeutic Implications and Targeting TG2 in CD

The central role of TG2 in gluten peptide modification, autoantigen formation, and amplification of HLA-restricted immune responses makes it an attractive therapeutic target in CD. Unlike the HLA genotype, which cannot be modified, TG2 represents a dynamic and potentially druggable molecular node positioned at the interface between environmental antigen exposure and adaptive immunity. Within the gene regulatory framework outlined in this review, therapeutic intervention can be conceptualized not only as inhibition of enzymatic activity but as modulation of the regulatory networks that govern TG2 expression and function.

### 8.1. Direct Inhibition of TG2 Enzymatic Activity

Pharmacological inhibition of TG2 aims to prevent deamidation of gliadin peptides, thereby reducing their affinity for HLA-DQ2 and HLA-DQ8 molecules and attenuating gluten-specific CD4^+^ T cell activation. Several small-molecule inhibitors have been developed, including compounds targeting the catalytic cysteine residue and inhibitors that interfere with calcium-dependent conformational activation of the enzyme, such as the selective active-site inhibitor ZED1227 [101,102].

Preclinical studies performed in cell-based systems and animal models indicate that selective TG2 inhibition can limit the generation of immunogenic epitopes while preserving essential physiological functions of the enzyme, including roles in wound healing, extracellular matrix stabilization, and intracellular signaling [63,103]. Achieving this functional selectivity is critical for therapeutic applicability.

Importantly, ZED1227 has been evaluated in human clinical trials, where oral administration during controlled gluten exposure resulted in a significant attenuation of gluten-induced mucosal damage and reduced immune activation markers [102]. These findings support the feasibility of targeting TG2 activity in patients with CD.

However, long-term efficacy, safety, and durability of response remain to be established in larger and more diverse patient populations [104]. In this context, direct enzymatic inhibition represents the most immediate and mechanistically targeted strategy to disrupt TG2-centered immune activation.

### 8.2. Modulation of Antigen Presentation and Immune Activation

Beyond direct inhibition of TG2, therapeutic strategies may target downstream steps of the pathogenic cascade, including antigen presentation and T cell activation. Approaches under investigation include peptide analogs designed to compete for HLA binding, tolerance-inducing immunotherapies targeting immunodominant gluten epitopes, and vaccine-based strategies aimed at reprogramming pathogenic T cell responses [105,106,107].

These strategies reflect the concept that CD results from the integration of biochemical peptide modification and adaptive immune amplification [108]. Consequently, targeting a single step of the cascade may not be sufficient for all patients, particularly in individuals with established immune memory [109,110]. From a systems perspective, effective intervention may require coordinated modulation of multiple nodes within the antigen-processing and immune activation network.

### 8.3. Targeting Inflammatory and Redox Pathways

Given the central role of inflammatory signaling and redox balance in regulating TG2 expression and activity, modulation of these pathways represents an indirect yet mechanistically coherent strategy to reduce gluten immunogenicity. Anti-inflammatory interventions that attenuate cytokine-driven *TGM2* transcription or suppress NF-κB activation may decrease local enzyme availability and limit substrate processing [87,89]. In parallel, restoration of redox homeostasis may influence TG2 activation by modulating the oxidative microenvironment of the intestinal mucosa, thereby affecting the conformational dynamics and catalytic competence of the enzyme [95,97,111].

In particular, these observations support a model in which TG2 activity is not solely determined by its expression level but emerges from the integration of inflammatory cues, redox-sensitive modifications, and microenvironmental constraints. Within this system, oxidative and inflammatory signals act as dynamic regulators that define spatial and temporal thresholds of TG2-mediated peptide deamidation, ultimately shaping antigen presentation and T cell activation.

Although antioxidant strategies alone are unlikely to reverse disease pathology, their integration into broader anti-inflammatory or immunomodulatory approaches may help restrain TG2-dependent enzymatic amplification under chronic inflammatory conditions [110,112]. This perspective shifts the therapeutic focus from isolated enzyme inhibition toward modulation of the regulatory microenvironment governing TG2 activity.

### 8.4. Precision Medicine and Stratified Risk Assessment

The integrated molecular perspective outlined in this review indicates that disease susceptibility arises from the convergence of HLA genotype, TG2 enzymatic activity, and regulatory modulation within the intestinal microenvironment [7,62]. This perspective supports the development of stratified therapeutic approaches based on combined genetic and molecular profiling [108].

Individuals with high-risk HLA configurations and increased mucosal *TGM2* expression may benefit most from targeted enzyme inhibition, whereas HLA-positive individuals with limited inflammatory activation may be effectively managed through dietary intervention alone [102,112].

Future studies integrating genetic, epigenetic, transcriptomic, single-cell, and proteomic data may enable the identification of molecular signatures predictive of disease progression and therapeutic responsiveness [41,92]. Such approaches may also clarify how TG2-centered regulatory networks translate environmental gluten exposure into coordinated immune responses, allowing risk assessment to move beyond a binary HLA-positive/HLA-negative classification toward a multidimensional model of disease susceptibility [110].

### 8.5. Future Directions

Although a strict gluten-free diet remains the cornerstone of disease management, it does not address the molecular mechanisms underlying immune activation. Targeting TG2 and its regulatory pathways therefore represents a strategy to intervene upstream in the pathogenic cascade [108].

Future approaches should combine enzymatic inhibition with modulation of transcriptional and epigenetic mechanisms controlling TG2 expression and activity. Such strategies may enable stage-specific intervention and improve therapeutic precision. Key challenges include preserving physiological TG2 functions, minimizing off-target effects, and defining the optimal timing of intervention. Longitudinal and stratified studies will be required to determine whether early modulation of TG2 activity can prevent disease onset in genetically predisposed individuals or limit disease progression after immune activation has been established [113,114].

Translation into clinical application will depend on integrating genetic predisposition, peptide modification, and gene regulatory dynamics into a coherent framework. In this context, TG2 should be considered a system-level target linking gene regulation to antigen processing and immune activation. Ultimately, effective therapeutic strategies will require a deeper understanding of how these processes converge to shape disease development at the individual level [115,116,117].

### 8.6. Limitations and Perspectives

This review proposes an integrated model linking TG2 activity to gene regulatory mechanisms in CD; however, several limitations must be considered. Direct experimental evidence connecting *TGM2* transcriptional regulation to antigen-specific immune activation in vivo remains limited. Current knowledge is largely based on indirect data, including transcriptomic analyses, in vitro studies, and extrapolation from related inflammatory conditions.

The proposed framework integrates heterogeneous data derived from multiple experimental systems and levels of resolution, ranging from bulk tissue analyses to single-cell approaches. While this integration provides a broad perspective, it may not fully capture disease-specific mechanisms across all clinical contexts.

In addition, the interplay between TG2 regulation, HLA-restricted antigen presentation, and microenvironmental factors is not yet fully defined at the mechanistic level, particularly with respect to spatial and temporal dynamics within the intestinal mucosa.

Further studies combining multi-omics approaches, including single-cell transcriptomics, epigenetic profiling, and functional validation, will be required to define causal relationships and refine the proposed model. These limitations do not undermine the framework but delineate the boundaries within which it should be interpreted and highlight key priorities for future investigation.

## 9. Conclusions

Overall, the available evidence supports a model in which TG2 links environmental gluten exposure to HLA-restricted adaptive immune responses. Rather than acting as an isolated enzymatic factor, TG2 integrates antigen processing with gene regulatory mechanisms that control its expression and activity within the intestinal mucosa. Within this framework, disease susceptibility reflects the interaction of three principal components: (i) HLA-dependent structural permissiveness; (ii) TG2-mediated biochemical modification of gluten peptides; and (iii) gene regulatory processes controlling *TGM2* expression and enzymatic activity.

Importantly, these findings position TG2 at the interface between gene regulation and antigen processing, highlighting transcriptional control of TG2 as a key determinant of HLA-restricted immunity in CD.

This perspective provides a mechanistic basis for inter-individual variability and supports the development of therapeutic strategies targeting TG2 and its regulatory network.

## Figures and Tables

**Table 1:** Multi-layered regulation of TG2 expression and activity in CD.

Regulatory Level	Mechanism	Effect on TG2	Relevance in CD	References
Conformational regulation	Ca^2+^ versus GTP binding	Switch between inactive and active conformations	Controls enzymatic activation and deamidation of gliadin peptides	[17,19]
Catalytic regulation	Competition between amine donors and water	Determines transamidation versus deamidation balance	Regulates generation of immunogenic epitopes	[15,37,49]
Redox regulation	Cys277 oxidation (disulfide formation, S-nitrosylation)	Reversible inhibition of catalytic activity	Links oxidative stress to antigen processing	[22,23]
Calcium signaling	Inflammation-driven Ca^2+^ increase	Stabilization of the active conformation	Enhances TG2 enzymatic activity	[7,45]
Cytokine signaling	IFN-γ, TNF-α, IL-1β, IL-15	Upregulation of *TGM2* transcription via NF-κB and JAK/STAT	Increases TG2 availability in inflamed tissues	[7,71,73,74]
NF-κB pathway	Inflammatory signaling cascades	Induction and amplification of *TGM2* expression	Couples immune activation to TG2 regulation	[24,73]
Cellular stress (UPR)	Gliadin-induced unfolded protein response	Increased TG2 expression and cytokine production	Connects epithelial stress to inflammation	[74]
Epigenetic regulation	DNA methylation and histone modifications	Modulation of *TGM2* transcriptional accessibility	Sustains inflammatory gene expression programs	[75,76,78,80]
Post-transcriptional regulation	microRNAs (e.g., miR-21, miR-155)	Regulation of mRNA stability and translation	Fine-tunes immune signaling pathways	[82]
Genetic variability	Regulatory SNPs	Modulation of gene expression responsiveness	Influences threshold for deamidation of gliadin peptides	[83,84]
Non-HLA genes	*IL2*, *IL21*, *SH2B3*, *TAGAP*, *CTLA4*	Modulation of immune signaling pathways	Affects antigen presentation and T cell activation	[60,61,85]
Barrier function	Increased intestinal permeability	Enhanced exposure of gliadin substrates to TG2	Promotes peptide modification	[47,74]
Microenvironment	Inflammation and ROS	Context-dependent modulation of TG2 activity	Shapes immune activation dynamics	[22,50,52]

**Table 2:** Major non-HLA genetic loci and their functional roles in CD.

Gene	Function	Pathway	Role in CD	References
*IL2/IL21*	Regulation of T cell proliferation and differentiation	Cytokine signaling (JAK/STAT)	Promotes expansion and effector differentiation of gluten-specific CD4^+^ T cells	[60,85]
*SH2B3*	Negative regulator of cytokine receptor signaling	JAK–STAT pathway	Limits cytokine signaling thresholds and modulates immune activation	[60,85]
*CTLA4*	Negative regulator of T cell activation	Immune checkpoint pathway	Maintains peripheral tolerance and restrains autoreactive T cell responses	[60,85]
*TAGAP*	Regulation of T cell receptor signaling and cytoskeletal dynamics	TCR signaling pathway	Controls immune synapse organization and T cell activation efficiency	[60,85]

## Data Availability

No new data were created or analyzed in this study. Data sharing is not applicable to this article.

## Abbreviations

The following abbreviations are used in this manuscript:

APC	Antigen-presenting cell
APCs	Antigen-presenting cells
CD	Celiac disease
HLA	Human leukocyte antigen
HLA-DQ2/DQ8	Major histocompatibility complex class II molecules associated with celiac disease
IELs	Intraepithelial lymphocytes
IFN-γ	Interferon gamma
IL-1β	Interleukin 1 beta
IL-15	Interleukin 15
JAK/STAT	Janus kinase/Signal transducer and activator of transcription pathway
MAPK	Mitogen-activated protein kinase
miRNA	microRNA
NF-κB	Nuclear factor kappa B
NKG2D	Natural killer group 2, member D
TG2	Transglutaminase 2
TG2-DG	TG2–deamidated gliadin
*TGM2*	Transglutaminase 2 gene
TNF-α	Tumor necrosis factor alpha
UPR	Unfolded protein response
